# Supplementary material for: Expression Patterns of Clock Gene mRNAs and Clock Proteins in Human Psoriatic Skin Samples
Source: Int J Mol Sci. 2021 Dec 23;23(1):121. doi: 10.3390/ijms23010121 (PMC8745255; doi:10.3390/ijms23010121)
Supplement: Supplementary file 1 [file ijms-23-00121-s001.zip › ijms-1483793-supplementary.pdf]

Table S1: The significance data in cytokine treated HaCaT cells compared to control.

	bmal1				***rev-erbα***				***per1***			
	**control *vs***				**control *vs***				**control *vs***			
**h**	**TNF-α**	**IL-22**	**IL-1β**	**IL-17A**	**TNF-α**	**IL-22**	**IL-1β**	**IL-17A**	**TNF-α**	**IL-22**	**IL-1β**	**IL-17A**
0	ns	ns	ns	ns	ns	ns	ns	ns	ns	ns	ns	ns
4	ns	ns	ns	0,0122	ns	ns	ns	ns	ns	ns	ns	ns
8	ns	ns	ns	0,0166	ns	ns	ns	ns	ns	ns	ns	ns
12	<0,0001	<0,0001	0,0098	<0,0001	ns	ns	0,0166	0,0039	ns	ns	ns	ns
16	<0,0001	<0,0001	0,0122	0,0002	0,0187	ns	<0,0001	<0,0001	ns	ns	ns	0,0276
20	<0,0001	ns	0,0010	0,0046	<0,0001	ns	ns	ns	ns	ns	ns	ns
24	<0,0001	ns	0,0012	<0,0001	0,0003	ns	0,0261	ns	ns	ns	ns	ns
28	<0,0001	ns	0,0005	ns	ns	ns	0,0119	ns	ns	ns	ns	ns
32	<0,0001	0,0059	ns	0,0216	ns	ns	0,0027	0,0357	ns	ns	ns	ns
36	<0,0001	<0,0001	ns	0,0030	0,0004	ns	0,0007	0,0063	ns	ns	ns	ns
40	<0,0001	ns	ns	ns	0,0001	ns	0,0035	ns	ns	ns	ns	ns
44	<0,0001	ns	ns	ns	0,0307	ns	0,0087	0,0162	ns	0,0216	ns	ns

	per2				***cry1***				***clock***			
	**control *vs***				**control *vs***				**control *vs***			
**h**	**TNF-α**	**IL-22**	**IL-1β**	**IL-17A**	**TNF-α**	**IL-22**	**IL-1β**	**IL-17A**	**TNF-α**	**IL-22**	**IL-1β**	**IL-17A**
0	ns	ns	ns	ns	ns	ns	ns	ns	ns	ns	ns	ns
4	ns	ns	ns	ns	ns	ns	ns	ns	ns	ns	ns	ns
8	ns	ns	ns	ns	ns	ns	ns	ns	ns	ns	ns	ns
12	ns	ns	ns	ns	ns	ns	ns	ns	ns	ns	ns	ns
16	ns	ns	ns	ns	ns	ns	ns	ns	ns	ns	ns	ns
20	ns	ns	ns	ns	ns	ns	ns	ns	ns	ns	ns	ns
24	ns	ns	ns	ns	ns	ns	ns	ns	ns	ns	ns	ns
28	ns	ns	ns	ns	ns	ns	ns	ns	ns	ns	ns	ns
32	ns	ns	ns	ns	ns	ns	ns	ns	ns	ns	ns	ns
36	ns	ns	ns	ns	ns	ns	ns	ns	ns	ns	ns	ns
40	ns	ns	ns	ns	ns	ns	ns	ns	ns	ns	ns	ns
44	ns	ns	ns	ns	ns	ns	ns	ns	ns	ns	ns	ns
